# Supplementary material for: Ultrafast shock synthesis of nanocarbon from a liquid precursor
Source: Nat Commun. 2020 Jan 17;11:353. doi: 10.1038/s41467-019-14034-z (PMC6968971; doi:10.1038/s41467-019-14034-z)
Supplement: Supplementary file 1 — Supplementary information [file 41467_2019_14034_MOESM1_ESM.pdf]

Supplementary information

Ultrafast shock synthesis of nanocarbon from a liquid precursor

Armstrong et al.

Supplementary Note 1

The shock Hugoniot of a material is the set of final thermodynamic equilibrium states that can be reached via shock compression from an initial state (p_0, v_0, e_0) (p_0 – pressure, v_0 – specific volume, e_0 – specific energy); it is usually represented as a $p(v)$ curve, with the corresponding energy given by the Hugoniot equation[1]. Shock Hugoniots are typically measured by impact or laser-driven piston experiments, where the sample is rapidly compressed by an incident surface moving at speed u_p, which generates a shock moving at speed u_s – see Supplementary Figure 1a. The experimentally measured u_p and u_s map to pressure

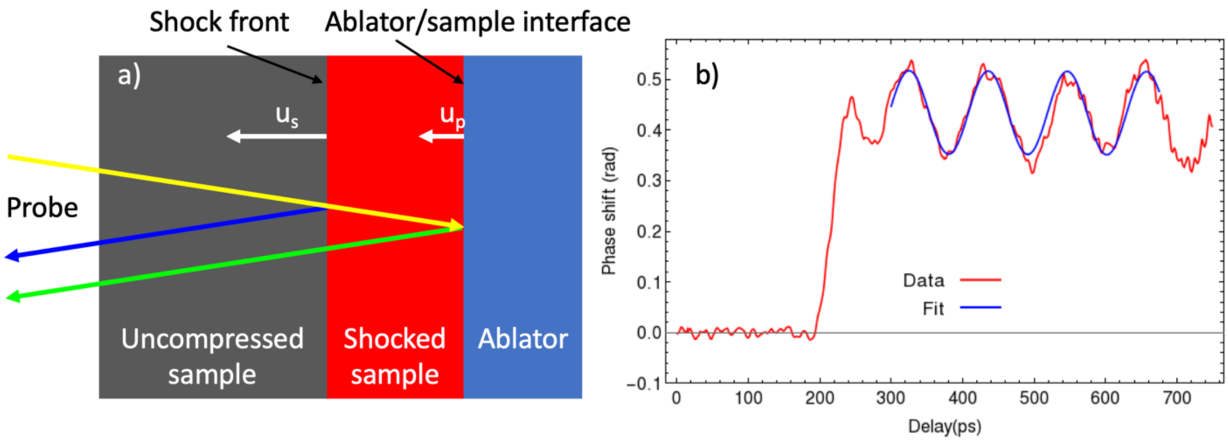

Supplementary Figure 1: Analysis of ultrafast experiments. a) Conceptual representation of experimental set-up: optical reflections from the moving shock front and the Al piston are Doppler-shifted to higher frequency resulting in a phase shift per unit time that beats due to the frequency difference between the piston and shock reflections. b) An example of data from the experiment; these data are fit to a model to extract shock and piston speeds. Source data are provided as a Source Data file.

and specific volume of the shocked state by mass and momentum conservation across the shock front. As a result, u_s vs. u_p plots are often also referred to as shock Hugoniots in the shock-wave literature. Please refer to Nellis[1] for more information about shock-wave compression and Armstrong et al.[2] for details about ultrafast compression and the generation and interpretation of data shown in Supplementary Figure 1b.

An important question for ultrafast shock experiments is whether compression on very short (≈1 ns) time scales obtains Hugoniots that are equivalent to longer time scale experiments, e.g. using gas gun driven impactors, which are known to generate final states over 100s of ns to μs. Substantial existing work[3–5] demonstrates that for materials which equilibrate rapidly prior to the onset of chemistry (e.g. liquids) or

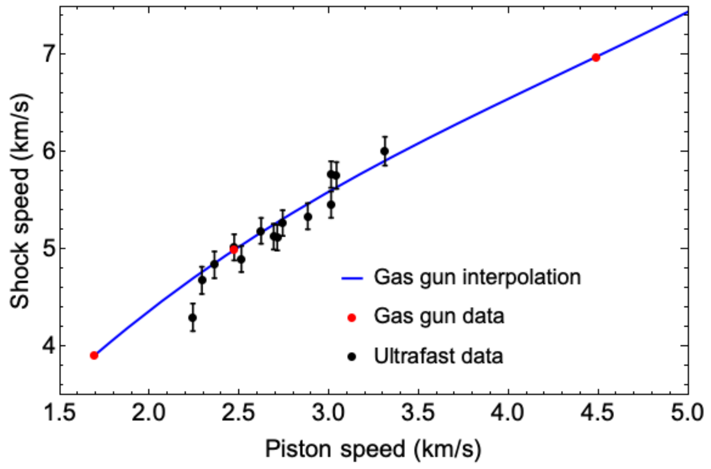

Supplementary Figure 2: Ultrafast shock Hugoniot data compared with gas gun data from Nellis *et al.* [6]. The line is a third order spline interpolation of the gas gun data (including points outside of the domain of the graph). Error bars are single prediction errors from a linear fit to the ultrafast data. Source data are provided as a Source Data file.

undergo very rapid chemistry, ultrafast compression does obtain an equivalent Hugoniot. A comparison of the gas-gun Hugoniot data of Nellis et al.[6] and the measured Hugoniot from this work at pressures below 16 GPa (see Supplementary Figure 2) demonstrates this here as well for liquid CO.

Supplementary Discussion

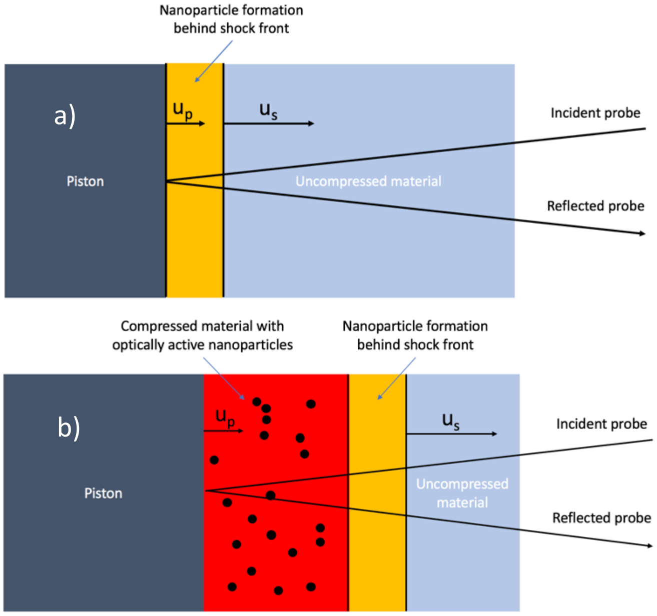

Supplementary Figure 3: Schematic showing shock-wave propagation in the sample at two times shortly after shock arrival. a) Early time, prior to formation of nanoparticles which exhibit the bulk dielectric response of liquid carbon, and b) Later time, when nanoparticles show liquid carbon dielectric response and have approximately constant volume fraction in a volume that expands with shock propagation.

Probe signal loss through the sample at shock pressures higher than ≈16 GPa is likely due to absorption and Rayleigh scattering caused by the precipitation of liquid carbon nanoparticles. Attenuation through Rayleigh scattering is however orders of magnitude smaller than from absorption for particle sizes in the nanometer range[7], so we neglect it here. To model probe absorption subsequent to shock compression we assume (as shown schematically in Supplementary Figure 3) that carbon nanoparticles form behind the shock front and grow to sufficient size to exhibit liquid carbon bulk dielectric response on a time scale of tens of ps. Thereafter, consistent with simulations, we assume that their volume fraction remains approximately constant at ≈10%, while the volume of compressed material grows with shock propagation, thus increasing the length of the nanoparticle-containing region through which probe light must pass. Consequently, the probe reflection from the piston (Al/sample) interface will be absorbed according to a variation of Beer's law: $I(t) = I_0 e^{-\alpha\, L(t)}$, where I_0 is the incident intensity, $I(t)$ is the intensity as a

function of time, α is the absorption constant, and $L(t)$ is the thickness of the absorbing material. (We neglect here the reflection from the shock front, which is typically much smaller than the piston reflection). We approximate the (time-dependent) thickness of the absorption layer by:

$$L(t) = 2(u_{\mathrm{s}} - u_{\mathrm{p}})(t - t_0), t > t_0 \tag{1}$$

As shown in Supplementary Figure 3 we assume the existence of a transition layer in which nascent carbon nanoparticles do not yet exhibit liquid carbon dielectric response. Fig. 2 (main text) shows that the absorption for shots above 16 GPa deviates from the lower pressure shots around t_0=50 ps after shock arrival – we postulate that this is when nanoparticles begin showing metallic character. Additionally, for u_{s}.vs.u_{p} we employ the interpolation shown in Supplementary Figure 2. With the above assumptions the calculated intensity as a function of time is shown in Fig. 2 (main text); the variation in absorption – light grey band, is due to uncertainty in the liquid carbon dielectric function (see below).

The absorption constant is given by:

$$\alpha = \rho_{\mathrm{n}} \sigma_{\mathrm{abs}} \tag{2}$$

, where ρ_{n} is the number density of nanoparticles and the absorption cross section is given by [7,8]:

$$\sigma_{\mathrm{abs}} = \frac{18\pi\, V\, \epsilon_{\mathrm{m}}^{3/2}}{\lambda} \frac{\epsilon_2}{|\epsilon + 2\epsilon_{\mathrm{m}}|^2} \tag{3}$$

Here V is the volume of a nanoparticle with dielectric function $\epsilon = \epsilon_1 + i\,\epsilon_2$; ϵ_{m} is the relative dielectric constant of the surrounding medium, and λ is the probe wavelength. This expression is valid when the nanoparticle diameter (d) is much smaller than λ, which should be well satisfied here since the simulations suggest that d =1-10 nm while recovered samples have characteristic particle sizes of 5-30 nm, and λ = 800 nm. Due to the particle volume dependence, the absorbance is constant for a fixed volume fraction of nanoparticles, thereby removing an explicit dependence on the nanoparticle size. To estimate σ_{abs} we employ the dielectric function of liquid carbon inferred from femtosecond spectroscopy[9] at conditions

similar with our experiment and estimate $\epsilon_{\mathrm{m}} = 2.6$ using the Clausius-Mossotti relation along with thermochemical calculations that yield the molecular composition (approximately equimolar mixture of CO and CO_2 with trace amounts of O_2) and density (≈2 g/cm^3) of the background fluid. This yields absorption depths α^{-1} of 380 nm and 514 nm, corresponding to the two possible liquid carbon dielectric functions proposed in Ref. 9; these two values generate the intensity decay grey band in Fig. 2 (main text).

The above analysis suggests that carbon nanoparticle formation lags only ≈50 ps behind the shock arrival in the sample, as indicated by the strong deviation of the reflectance from the Al background. This is likely an upper bound, since smaller particles may exist at earlier times, but not exhibit sufficiently metallic behavior to affect the absorption.

Supplementary Note 2

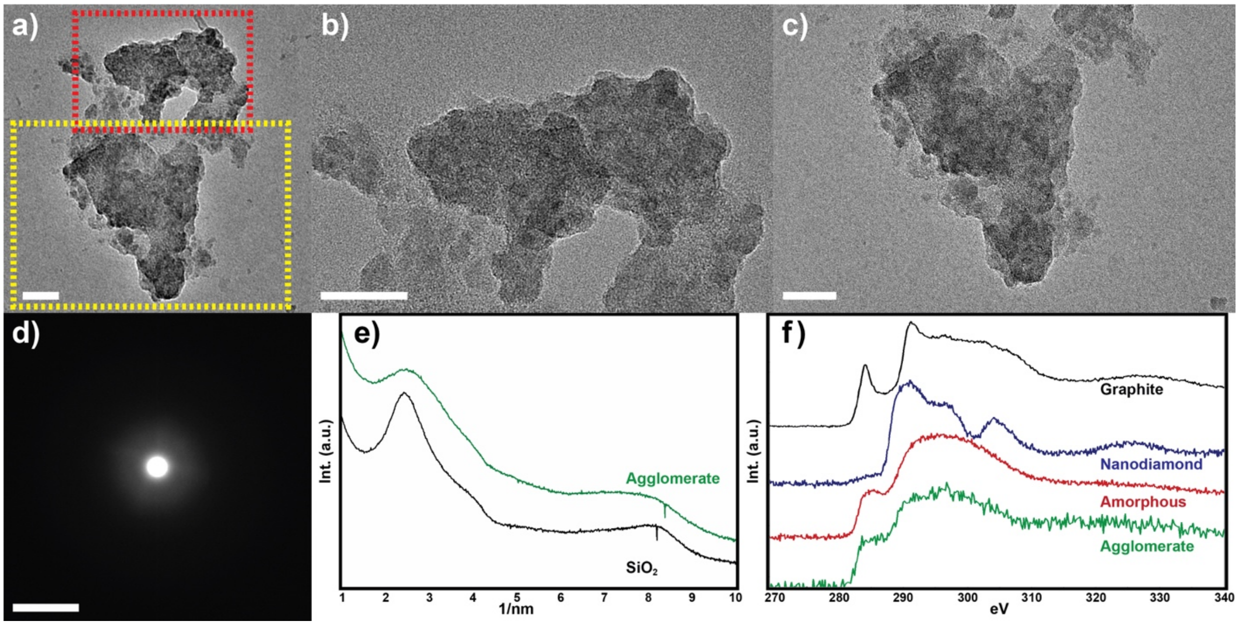

Supplementary Figure 4: TEM analysis of carbonaceous agglomerate. Defocused image of representative network of agglomerated globular particles a) on SiO_2 membrane highlights particulate constituents. Higher magnification images of red b) and yellow c) boxed regions in a). Diffraction signal d) from agglomerate region in c) and radial integrations e) of d) and diffraction signal from bare SiO_2 membrane. Diffraction pattern from agglomerate is consistent with amorphous carbon. Carbon K edge EELS spectra f) from agglomerate in a) and reference carbon materials ("Amorphous" corresponds to the lacey carbon of TEM grids. Agglomerate spectrum matches well that of amorphous carbon. Scale bars are 50 nm (a – c) and 5 1/nm d). Source data are provided as a Source Data file.

Imaging, diffraction, and EELS data for a single agglomerate are shown in Supplementary Figure 4. As with the agglomerates imaged in Fig. 3 (main text), roughly spherical globular domains are observed throughout the agglomerated structure. Radial integrations of diffraction patterns from the agglomerate on an underlying amorphous SiO_2 membrane as well as a bare region of the same membrane are compared. The broad, weak peaks visible in the sample signal are consistent with amorphous carbon. A carbon K edge EELS spectrum of the agglomerate region displayed in Supplementary Figure 4c (this panel is the same as Figure 3c in the main manuscript) is compared with reference spectra from graphite, nanodiamond, and amorphous carbon, and is a close match to the amorphous carbon reference.

For the sake of completeness, we also include below a comparison of the original and marked TEM images shown in Fig. 3a, b (main text).

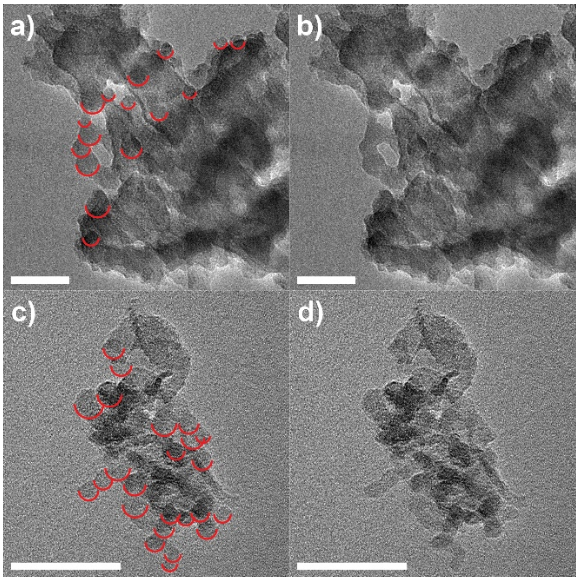

Supplementary Figure 5: Side by side comparison of the marked and original TEM images of carbon agglomerates shown in Fig. 3a, b (main text). Scale bars are 50 nm.

Supplementary Note 3

The resulting Raman spectrum shows bands representative of disordered carbon - both the D and G bands are observed at 1354 and 1566 cm^{-1}, respectively – see Supplementary Figure 6; no 2D+G band is present at +2500cm^{-1}. Comparisons of this Raman spectrum with the published spectra of various forms of carbon suggest that the solid residue is predominantly amorphous carbon, with possible nanodiamond content. Previous studies have shown that the G-band is associated with the presence of sp^2 bonds, while the D-band points to structural disorder and/or sp^3 bonding[10–15]. We also note the presence of a low intensity peak at 1190 cm^{-1}, which was previously observed in the spectrum of nanocrystalline diamond[15]. Besides the carbon Raman bands we also observed a broad Raman background at high frequencies, which is likely due primarily to amorphous nanocarbon surface effects. The origin of the sharp Raman modes at lower frequencies (below 1000 cm^{-1}) is not entirely clear at this time. Al_2O_3 and/or Al_4C_3 could plausibly form due to the Al ablator used in these experiments. The observed Raman modes are not due to Al_2O_3 and we could only partially, not conclusively, assign them to Al_4C_3. It is worth noting that neither Al_2O_3 nor Al_4C_3 have Raman peaks close to the 1354 cm^{-1} (carbon D band) peak.

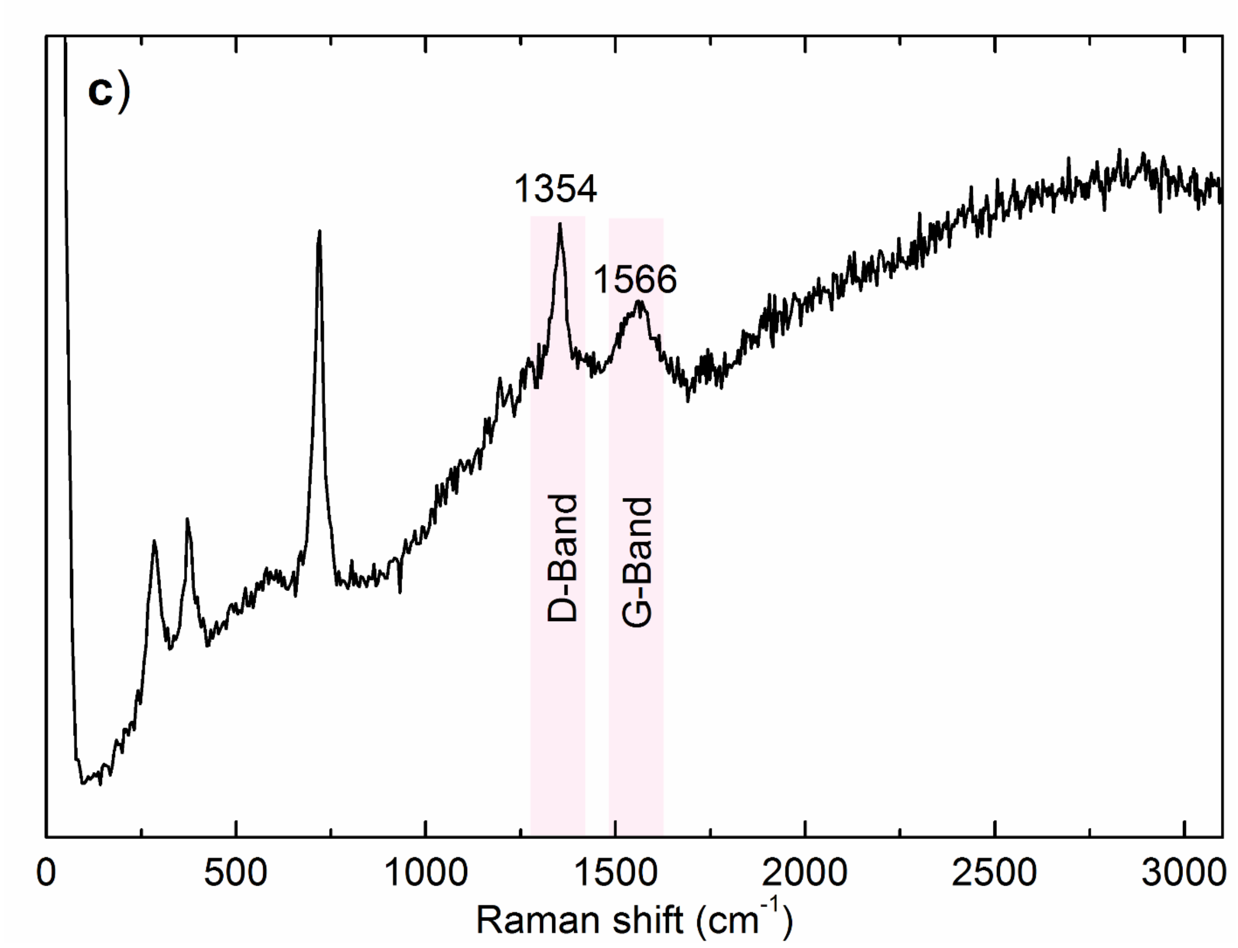

Supplementary Figure 6: Room temperature Raman spectrum after experiment completion, prior to opening the cell, showing amorphous carbon Raman bands. Source data are provided as a Source Data file.

Supplementary Note 4

Cluster diameters (up to ≈ 4nm) found in the present 0.25ns simulations (which were completed prior to analysis of the experimental results) are consistent with the experimental cluster (nanoparticle) sizes (5 - 30 nm) obtained from the TEM imaging of recovered carbon condensates. We show in Supplementary Figure 7 a typical cluster, along with a detail of its oxygen-decorated surface. The clusters are immersed in a sea of small molecular species with very short lifetimes[16]; this is in line with previous observations of high pressure - high temperature chemistry in the fluid phase[17]. Future simulation studies will focus on examining the influence of the C:O ratio and P/T conditions on condensate formation and the effects of the thermodynamic quenching pathway on the nanoparticles' composition, structure and morphology.

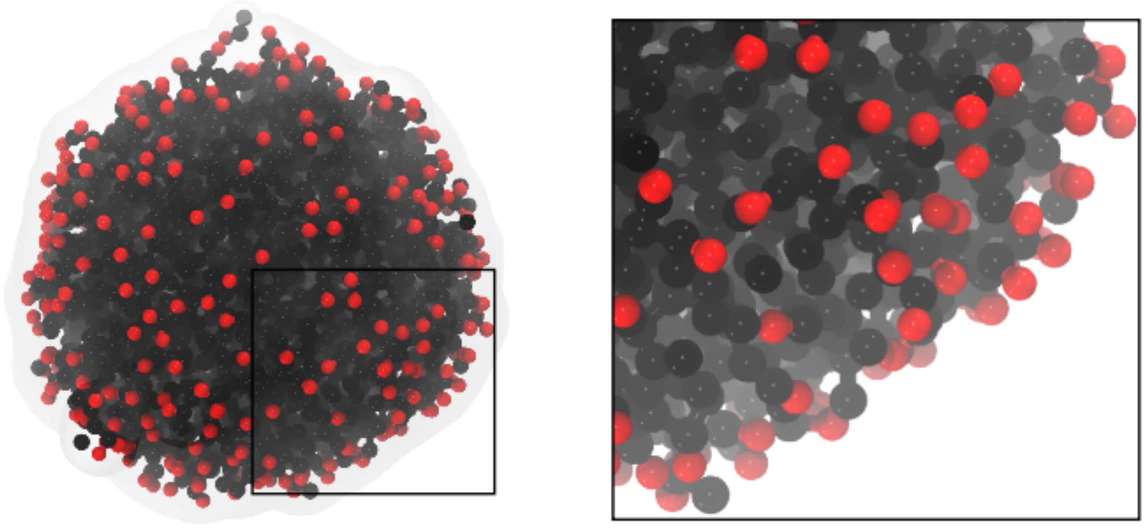

Supplementary Figure 7: Typical carbon cluster and surface detail indicating oxygen (red) enrichment.

Supplementary references

1. Nellis, W. J. Dynamic compression of materials: metallization of fluid hydrogen at high pressures. *Rep. Prog. Phys.* **69**, 1479–1580 (2006).
2. Armstrong, M. R., Crowhurst, J. C., Bastea, S. & Zaug, J. M. Ultrafast observation of shocked states in a precompressed material. *J. Appl. Phys.* **108**, 023511 (2010).

3. Armstrong, M. R., Zaug, J. M., Grant, C. D., Crowhurst, J. C. & Bastea, S. Ultrafast Shock Compression of an Oxygen-Balanced Mixture of Nitromethane and Hydrogen Peroxide. *J. Phys. Chem. A* **118**, 6148–6153 (2014).

4. Armstrong, M. R. *et al.* Ultrafast Shock Initiation of Exothermic Chemistry in Hydrogen Peroxide. *J Phys Chem A* **117**, 13051–13058 (2013).

5. Brown, K. E., McGrane, S. D., Bolme, C. A. & Moore, D. S. Ultrafast Chemical Reactions in Shocked Nitromethane Probed with Dynamic Ellipsometry and Transient Absorption Spectroscopy. *J Phys Chem A* **118**, 2559–2567 (2014).

6. Nellis, W. J., Ree, F. H., van Thiel, M. & Mitchell, A. C. Shock compression of liquid carbon monoxide and methane to 90 GPa (900 kbar). *J. Chem. Phys.* **75**, 3055–3063 (1981).

7. Arbouet, A. *et al.* Direct measurement of the single-metal-cluster optical absorption. *Phys. Rev. Lett.* **93**, 127401 (2004).

8. Hartland, G. V. Optical Studies of Dynamics in Noble Metal Nanostructures. *Chem. Rev.* **111**, 3858–3887 (2011).

9. Reitze, D. H., Ahn, H. & Downer, M. C. Optical properties of liquid carbon measured by femtosecond spectroscopy. *Phys. Rev. B* **45**, 2677–2693 (1992).

10. Ferrari, A. C. & Robertson, J. Interpretation of Raman spectra of disordered and amorphous carbon. *Phys. Rev. B* **61**, 14095–14107 (2000).

11. Ferrari, A. C. & Robertson, J. Resonant Raman spectroscopy of disordered, amorphous, and diamondlike carbon. *Phys. Rev. B* **64**, 075414 (2001).

12. Shang, N. G., Silva, S. R. P., Jiang, X. & Papakonstantinou, P. Directly observable G band splitting in Raman spectra from individual tubular graphite cones. *Carbon* **49**, 3048–3054 (2011).

13. Malard, L. M., Pimenta, M. A., Dresselhaus, G. & Dresselhaus, M. S. Raman spectroscopy in graphene. *Phys. Rep.* **473**, 51–87 (2009).

14. Chu, P. K. & Li, L. Characterization of amorphous and nanocrystalline carbon films. *Mater. Chem. Phys.* **96**, 253–277 (2006).

15. Prawer, S. *et al.* The Raman spectrum of nanocrystalline diamond. *Chem. Phys. Lett.* **332**, 93–97 (2000).

16. Lindsey, R. K., Goldman, N., Fried, L. E. & Bastea, S. Development of the ChIMES Force-Field for Reactive Molecular Systems: Carbon Monoxide at Extreme Conditions. Preprint at https://chemrxiv.org/s/e482138dc3fccfa55ee9. (2019).

17. Wu, C. J., Fried, L. E., Yang, L. H., Goldman, N. & Bastea, S. Catalytic behaviour of dense hot water. *Nat. Chem.* **1**, 57–62 (2009).
